# PPARγ2 Regulates a Molecular Signature of Marrow Mesenchymal Stem Cells

**DOI:** 10.1155/2007/81219

**Published:** 2007-08-27

**Authors:** K. R. Shockley, C. J. Rosen, G. A. Churchill, B. Lecka-Czernik

**Affiliations:** ^1^The Jackson Laboratory, 600 Main Street, Bar Harbor, ME 04609, USA; ^2^Department of Orthopaedic Surgery, Center for Diabetes and Endocrine Research, University of Toledo Medical Center, 3035 Arlington Avenue, Mail Stop 1008, Toledo, OH 43614, USA

## Abstract

Bone formation and hematopoiesis are anatomically juxtaposed and share common regulatory mechanisms. Bone marrow mesenchymal stromal/stem cells (MSC) contain a compartment that provides progeny with bone forming osteoblasts and fat laden adipocytes as well as fibroblasts, chondrocytes, and muscle cells. In addition, marrow MSC provide an environment for support of hematopoiesis, including the development of bone resorbing osteoclasts. The PPARγ2 nuclear receptor is an adipocyte-specific transcription factor that controls marrow MSC lineage allocation toward adipocytes and osteoblasts. Increased expression of PPARγ2 with aging correlates with changes in the MSC status in respect to both their intrinsic differentiation potential and production of signaling molecules that contribute to the formation of a specific marrow micro-environment. Here, we investigated the effect of PPARγ2 on MSC molecular signature in respect to the expression of gene markers associated exclusively with stem cell phenotype, as well as genes involved in the formation of a stem cell supporting marrow environment. We found that PPARγ2 is a powerful modulator of stem cell-related gene expression. In general, PPARγ2 affects the expression of genes specific for the maintenance of stem cell phenotype, including LIF, LIF receptor, Kit ligand, SDF-1, Rex-1/Zfp42, and Oct-4. Moreover, the antidiabetic PPARγ agonist TZD rosiglitazone specifically affects the expression of “stemness” genes, including ABCG2, Egfr, and CD44. Our data indicate that aging and anti-diabetic TZD therapy may affect mesenchymal stem cell phenotype through modulation of PPARγ2 activity. These observations may have important therapeutic consequences and indicate a need for more detailed studies of PPARγ2 role in stem cell biology.

## 1. INTRODUCTION

PPARγ, an essential regulator of lipid, glucose, and insulin metabolism [1], is expressed in bone marrow mesenchymal stem cells (MSC). PPARγ is expressed in mice and humans in two isoforms, PPARγ1 and PPARγ2, which originate from up to seven different transcripts due to alternative promoter usage and alternative splicing [2–5]. PPARγ2 differs from PPARγ1 by 30 additional amino acids on its N-terminus, which constitute AF-1 domain of ligand-independent gene-activating function [6]. While PPARγ1 is expressed in a variety of cell types, including osteoblasts, PPARγ2 is expressed in cells of adipocyte lineage and serves as an essential regulator of adipocyte differentiation and function [7, 8].

Osteoblasts and adipocytes are derived from a marrow mesenchymal cell compartment which also serves as a source of progenitors for marrow fibroblasts and cartilage cells and functions as hematopoiesis-supporting stroma [9, 10]. Commitment of marrow MSC toward adipocyte and osteoblast lineage occurs by a stochastic mechanism, in which lineage-specific transcription factors (such as Runx2 for osteoblasts and PPARγ2 for adipocytes) representing intrinsic determinants of this process are activated [8, 11]. Embryonic stem cells with a null mutation in PPARγ spontaneously differentiate to osteoblasts and are unable to differentiate to adipocytes [12]. In marrow MSC, PPARγ2 acts as a dominant negative regulator of osteoblast differentiation [8, 13]. Using a model of marrow MSC differentiation (U-33/γ2 cells), we have previously demonstrated that activation of the PPARγ2 isoform by the highly specific agonist and antidiabetic thiazolidinedione (TZD), rosiglitazone, converted cells of osteoblast lineage to terminally differentiated adipocytes and irreversibly suppressed both the osteoblast phenotype and the osteoblast-specific gene expression [8]. The expression of PPARγ2 in marrow MSC increases with aging [14]. Moreover, bone marrow derived from old animals produces unknown PPARγ activator(s) that stimulates adipocyte differentiation and suppresses osteoblast differentiation [14]. These changes cause alterations in the milieu of intrinsic and extrinsic signals that determine MSC lineage allocation. For instance, this contributes to the preferential MSC differentiation toward adipocytes and decreased differentiation toward osteoblasts that leads to the development of senile osteopenia.

PPARγ plays an important role in the maintenance of bone homeostasis as demonstrated in several animal models of either bone accrual or bone loss depending on the status of PPARγ activity [12 15–19]. A decrease in PPARγ activity resulted in increased bone mass due to increased osteoblast number [12, 18], whereas increased PPARγ activity due to TZD administration led to the bone loss [15–17,19]. TZD-induced bone loss was accompanied with changes in the cellular composition of the bone marrow, such as decreased numbers of osteoblasts and increased numbers of adipocytes, and changes in the MSC phenotype characterized by a loss of MSC plasticity. These changes are characteristics for aging bone marrow [20]. Recently, several human studies have demonstrated that TZD use is associated with decreased bone mineral density and an increased risk of fractures in postmenopausal diabetic women [21–23]. This prompted US Food and Drug Administration to issue a warning of possible adverse effects of TZD on human bone.

The development of high throughput analysis of gene expression using microarrays has advanced studies on genes and signaling pathways controlled by a single gene product. The transcriptional role of PPARγ in either differentiated cells or functional tissues has been studied using DNA microarrays, mostly to determine its role in the physiology during disease and as a result of therapeutic treatment with TZDs of these target tissues [24–26]. None of these studies, however, were designed to test for the effect of the PPARγ2 isoform on the molecular signature of MSC. Using a model of marrow MSC differentiation under the control of the PPARγ2 transcription factor, we found that both the presence of PPARγ2 and its activation with the antidiabetic TZD, rosiglitazone, resulted in gene expression changes for multiple genes that characterize the stem cell phenotype and their phenotypic lineages. Even though our model was originally developed to study the mechanisms by which PPARγ2 suppressed osteoblastogenesis and promoted adipogenesis, our studies suggest that PPARγ2 has a profound effect on the expression of signature genes for cell “stemness.”

## 2. MATERIAL AND METHODS

### 2.1. Cell cultures and RNA isolation

Murine marrow-derived U-33 (previously referred to as UAMS-33) cells represent a clonal cell line spontaneously immortalized in the long term bone marrow culture conditions. To study the effect of PPARγ2 on marrow mesenchymal stem cell differentiation, U-33 cells were stably transfected with either PPARγ2 expression construct (referred to as U-33/γ2 cells) or an empty vector control (referred to as U-33/c cells) as described previously [8]. Several independent clones were retrieved after transfection and carefully analyzed for their phenotype. Clone 28.6, representing U-33/γ2 cells, and clone γc2, representing U-33/c cells, were used in the experiments presented in this manuscript. Cells were maintained in *α*MEM supplemented with 10% FBS heat-inactivated (Hyclone, Logan, UT), 0.5 mg/ml G418 for positive selection of transfected cells, 100 U/ml penicillin, 100 *μ*g/ml streptomycin, and 0.25 *μ*g/ml amphotericin (sigma) at 37°C in a humidified atmosphere containing 5% CO_2_. Media and additives were purchased from Life Technologies (Gaithersburg, MD).

Cells were propagated for one passage and than seeded at the density of $3\times 10^{5}$ cells/cm^2^. After 48 hours of growth, when cultures achieved approximately 80% confluency, cells were treated with either 1 *μ*M rosiglitazone or the same volume of vehicle (DMSO) for 2, 24, and 72 hours, followed by RNA isolation using RNeasy kit (QIAGEN Inc., Valencia, CA). The replicate experiment was performed independently on a fresh batch of cells. Two replicates were used for microarray analysis. The factorial design of experiment was 2×3×2 which corresponded to two cell lines (with and without PPARγ2), three time points (2, 24, 72 hours), and two treatment regiments (rosiglitazone and vehicle).

### 2.2. Microarray experiments

RNA quality was assessed using the Agilent Model 2100 Bioanalyzer (Agilent Technologies, Palo Alto, CA). Five micrograms of total RNA were processed for use on the microarray by using the Affymetrix GeneChip one-cycle target labeling kit (Affymetrix, Inc., Santa Clara, CA) according to the manufacturer's recommended protocols. The resultant biotinylated cRNA was fragmented then hybridized to the GeneChip Mouse Genome 430 2.0 Array (45,000 probe sets used to analyze over 39,000 mouse transcripts and variants from over 34,000 well-characterized mouse genes; Affymetrix, Inc.). The arrays were washed, stained, and scanned using the Affymetrix Model 450 Fluidics Station and Affymetrix Model 3000 scanner using the manufacturer's recommended protocols by the University of Iowa DNA Core Facility. Raw gene expression measurements were generated using the microarray suite (MAS) version 5.0 software (Affymetrix, Inc.). Statistical assessment of differential gene expression is described in Lecka-Czernik et al. [27].

## 3. RESULTS AND DISCUSSION

An essential role of PPARγ2 in the regulation of marrow MSC lineage allocation, together with the evidence of its increased activity in MSC with aging [14], prompted us to study the effect of PPARγ2 on the expression of stem cell gene markers. Two aspects were examined: the effect of the presence of PPARγ2 in U-33 stem cells and the effect of PPARγ2 activation with rosiglitazone on stem cell phenotype.

Here we used a model of marrow MSC differentiation under the exclusive control of a single protein, PPARγ2. This system allows for relatively unambiguous studies of the unique effects of PPARγ2 isoform on MSC phenotype. The model of PPARγ2-dependent MSC differentiation consists of two cell lines derived from the same parental cell line (U-33 cells), which either express the PPARγ2 protein (U-33/γ2 cells) or do not express the PPARγ2 protein (U-33/c cells) [8, 29]. To assess the effects of the presence of PPARγ2 on the phenotype of U-33 cells in nontreated conditions, we compared gene expression in U-33/γ2 and U-33/c cells maintained in basal growth conditions (this is referred to as the “P versus V” analysis). This comparison provides information about PPARγ2 activities, which are either ligand independent or acquired as a result of activation with natural ligands present in the growth media or endogenously produced by tested cells. The results of “P versus V” analysis may provide information on a role of PPARγ2 in a continuum of changes that occur in stem cells during aging. To assess an effect of rosiglitazone on the expression of stem cell-related genes, we compared gene expression in U-33/γ2 cells treated with rosiglitazone and nontreated U-33/γ2 cells (this is referred to as the “PR versus P” analysis). This analysis provides important information on the effects of rosiglitazone on the stem cell phenotype. Finally, comparison of the results of both analyzes provides information on differences between endogenous and artificially induced PPARγ2 activities in respect to stem cell gene expression.

To avoid differences in the cell phenotype due to different rates of cell growth, we chose the 72-hour time point for the analysis of gene expression (see Section 2). In basal growth conditions at this time point, cell cultures of U-33/γ2 and U-33/c were in state of confluence, cells acquired fibroblast-like appearance and cell cultures were indistinguishable morphologically from each other. In contrast, U-33/γ2 cells treated for 72 hours with rosiglitazone acquired adipocyte phenotype typified by large fat droplets. A morphological appearance of U-33/c cells treated with rosiglitazone was indistinguishable from nontreated U-33/c cells as well as nontreated U-33/γ2 cells.

There are no known exclusive markers for MSC. However, based on extensive work with MSCs and other stem cell populations, several proteins have emerged as candidate markers associated with a stem cell phenotype. These entities include ATP-binding cassette g2 (Abcg2), cell surface antigen CD44, stem cell factor or kit ligand (SCF/Kitl), epidermal growth factor receptor (Egfr), early growth response factor 2 (Egr2), leukemia inhibitory factor (Lif), leukemia inhibitory factor receptor (Lifr), and stromal-derived factor/CXC- chemokine ligand 12 (SDF-1/CXCL12). Based on the available published information for stem cell gene expression for the analysis, we arbitrarily chose 135 genes that represent markers of either early or lineage committed stem cells [9 30–34]. The analysis showed that the expression of 38% of analyzed genes was not affected by activation state of PPARγ2 (see Table 4), the expression of 28% genes was exclusively affected by the presence of PPARγ2 (“P versus V” analysis) (see Table 1(a)), and the expression of 10% genes was exclusively affected by rosiglitazone-activated PPARγ2 (“PR versus P” analysis) (see Table 1(b)). The genes whose expression was affected by both rosiglitazone-activated and nonactivated PPARγ2 constituted 24% of the total genes studied; their expression was affected in equal proportion either similarly (see Table 2) or in the opposite direction in these two conditions (see Table 3).

Comparison of the two cell lines indicates that a majority of analyzed genes are up-regulated in U-33/γ2 versus U-33/c cells (see Tables 1(a) and 3). Most of these genes are characteristic for stem cells of hematopoietic and neural lineages while some of them are expected to be up regulated in hematopoiesis supporting stromal cells (e.g. Kitl, RANKL (Table 1(a)), and the CXCL family (Tables 1(a) and 3)).

These interesting observations have at least two reasonable interpretations. The first interpretation suggests that observed differences are a reflection of different phenotypes of the two individual parental cells from which each of the two clones originated. Hence, differences in gene expression between both cell lines are PPARγ2-independent. The second possibility suggests that these differences are PPARγ2-dependent and result from either PPARγ2 ligand-independent activity or activity acquired from endogenous ligand. Several lines of evidence suggest a correlation between the adipocyte-like phenotype of marrow stroma cells and support for hematopoiesis [35, 36]. Hematopoiesis depends heavily on the microenvironment provided by mesenchymal cell compartment in the marrow and the ability of these cells to produce growth factors and cytokines that act in a paracrine fashion to influence the differentiation of hematopoietic progenitors. In the long term bone marrow cultures, an *in vitro* system of hematopoietic cell differentiation, stroma cell support for myelopoiesis, is provided by cultures consisting mostly of adipocytes [35, 37]. Similarly, *in vivo* studies in a model of SAMP6 mice that are characterized by senile osteopenia due to a diminished number of osteoblasts and increased myelopoiesis, correlates positively with an increased number of marrow adipocytes [38]. Interestingly, U-33/γ2 cells support osteoclastogenesis much better than U-33/c cells (unpublished observation), in part due to relatively higher RANKL (9-fold in “P versus V,” Table 3) and lower OPG (−34.6-fold in “P versus V”; Table 1(a)) expression. Another important regulator of bone marrow hematopoiesis, including osteoclastogenesis, is represented by the chemokine CXCL12 or SDF-1 [39, 40]. Growing experimental evidence indicates that CXCL12 and its receptor CXCR4 axis is not only required for hematopoietic stem cell signaling but also has a crucial role in the formation of multiple organ systems during embryogenesis as well as adult nonhematopoietic tissue regeneration and tumorigenesis [39]. According to our analysis, an expression of CXCL12, but not CXCR4, is up regulated in U-33/γ2 cells (“P versus V”) and suppressed by PPARγ2-activated with rosiglitazone (“PR versus P”) (see Table 3). Thus, it is conceivable that mesenchymal cells which express PPARγ2 acquire the adipocyte-like phenotype typified by the production of number of cytokines and support hematopoietic stem cell differentiation.

While PPARγ2 has a positive effect on the stromal phenotype supporting hematopoiesis, it has a negative effect on the expression of “stemness” genes. The expression of LIF cytokine and its receptor, a regulatory system required for the stem cell self renewal, is significantly suppressed in U-33/γ2 cells as compared to U-33/c cells (see Table 1(a)). Interestingly, activation of PPARγ2 with rosiglitazone did not affect the expression of these genes. The presence of PPARγ2 in U-33/γ2 cells suppresses the expression of Egr2/Krox20, a stem cell-specific transcription factor with a role in the development of nervous system and endochondrial bone formation [41]. Egr2/Krox20 also regulates osteoblast differentiation and osteocalcin expression [42]. Again, rosiglitazone does not affect Egr2/Krox20 gene expression (see Table 1(a)). PPARγ2 cellular presence also affects expression of Zfp42 transcription factor, which is a marker of human and murine embryonic stem (ES) cells. Expression of Zfp42 is down regulated during ES cell differentiation [43]. An artificial knockdown of Zfp42 with RNAi resulted in spontaneous differentiation of ES cells toward endoderm and mesoderm lineages, whereas its overexpression led to the loss of self-renewal capacity of ES cells [44].

The expression of ABCG2, a well recognized stem cell marker [45], was down-regulated in “PR versus P” (−3.1 fold) (see Table 1(b)) and slightly in “P versus V” (−1.3 fold, P<.01) conditions (not shown). ABCG2 represents an ATP-binding cassette (ABC) transporter which serves to efflux certain xenobiotics (including anticancer drugs) that can lead to the development of multidrug resistance syndrome. This is a significant obstacle in cancer treatment [46]. This gene is also considered to be a marker of primitive pluripotent stem cells, termed “side population,” which were identified based on their ability to exclude Hoest dye [45]. The ability to exclude a variety of substances may comprise a mechanism that protects stem cells from exogeneous and endogeneous toxins. Finding that ABCG2 expression is down regulated by PPARγ2, especially after activation with rosiglitazone, implicates PPARγ2 as a negative regulator of stem cell phenotype as well as a negative regulator of multidrug resistance. Similarly, Egfr a marker of early stem cells is down regulated by PPARγ2 when activated with rosiglitazone [47].

Interestingly, however, the expressions of Oct-4 (POU5f1) and FGF4, well recognized embryonic stem cell markers highly expressed in the totipotent and pluripotent ES cells [48, 49] are up regulated in U-33/γ2 cells compared to U-33/c cells and are not affected in U-33/γ2 cells treated with rosiglitazone (see Table 1(a)).

Another interesting grouping consists of genes whose expression is differentially regulated by both activated and nonactivated PPARγ2 (see Table 2). A number of genes implicated in early stem cell maintenance and recruitment, among them CD44, H2-D1, PCNA, CD109, Spred1 and 2, and Stag1 and 2, are down regulated in U-33/γ2 cells in both basal conditions and upon rosiglitazone treatment.

The last category represents gene markers specific for terminally-differentiated cells. Consistent with the proadipocytic and antiosteoblastic activities of PPARγ2 activated with rosiglitazone, the expression of the gene encoding FABP4 increases, whereas an expression of the gene underlying alkaline phosphatase decreases. Markers of the neuronal phenotype are either decreased (S100b, Table 2) or not affected (nestin and NCAMs, Table 4), and the expression of CD34, a bona fide marker for cells of hematopoietic lineage, is not affected (see Table 4). However, the expression patterns of gene markers characteristic for embryonic stem cells and a large number of markers that are associated with a nonmesenchymal phenotype, including markers of different hematopoietic and neuronal lineages, indicates that marrow mesenchymal U-33 cells possess a mixed phenotype with some characteristics of early primitive pluripotent stem cells and lineage oriented mesenchymal cells.

In conclusion, PPARγ2 is a powerful modulator of the stem cell phenotype and its activation with antidiabetic TZDs affect the expression of “stemness” genes. It is unclear at this time whether, and to what extent, PPARy2 is expressed in stem cells *in vivo* and whether this key transcription factor plays a significant role in stem cell biology. However, the findings presented here, together with previously published evidence of increased PPARγ2 expression in MSCs with aging [14] and a loss of marrow MSC plasticity or ability to convert between phenotypes as a result of aging and TZD therapy [20], suggest that aging and TZD therapy may affect stem cell phenotype through modulation of PPARγ2 activity. These observations may also have important therapeutic consequences and indicate a need for more detailed studies of PPARγ2 role in stem cell biology.

## Figures and Tables

**Table 1(a):** Genes expressed differently in P versus V.

Gene symbol	Probe ID^a^	FC^b^	Gene description	Biological process^c^
Cd3g	1419178_at	1.5	CD3 antigen, gamma polypeptide	Immune and hematopoietic system, cell surface receptor linked signal transduction

Cd3e	1445748_at	1.5	CD3 antigen, epsilon polypeptide	Cell surface receptor linked signal transduction, positive regulation of T cell proliferation and T cell receptor signaling pathway

Cd4	1419696_at	1.5	CD4 antigen	Immune response, cell adhesion, cell surface receptor linked signal transduction, positive regulation of T cell activation

Cd7	1419711_at	1.5	CD7 antigen	Immune response, myeloid cells antigen

Cd8a	1451673_at	1.7	CD8 antigen, alpha chain	Immune response, cell surface receptor linked signal transduction, cellular defense response, cytotoxic T cell differentiation

Cd19	1450570_a_at	1.9	CD19 antigen	Lymphocyte progenitors

Cd24a	1416034_at	9.8	CD24a antigen	Cell surface antigen expressed in T and B lymphocytes, macrophages, dendritic endothelial, and epithelial cells

Cd33	1450513_at	1.5	CD33 antigen	Myeloid cells antigen, cell adhesion

Cd37	1419206_at	1.7	CD37 antigen	B and T cell antigen

Cd96	1419226_at	1.5	CD96 antigen	T-cell activation, cell adhesion

Cd207	1425243_at	1.5	CD 207 antigen	Specific for Langerhans cell precursors

Cd209b	1426157_a_at	1.7	CD209b antigen	Dendritic cell-specific, positive regulation of tumor necrosis factor-alpha biosynthesis, positive regulation of phagocytosis

Cd209c	1421562_at	1.9	CD209c antigen	Dendritic cell specific

Cxcl9	1418652_at	1.6	Chemokine (C-X-C motif) ligand 9	Inflammatory response, immune response

Cxcl13	1448859_at	2.0	Chemokine (C-X-C motif) ligand 13	Chemotaxis, inflammatory response, immune response, lymph node development

Cxcl16	1418718_at	1.7	Chemokine (C-X-C motif) ligand 16	Chemotaxis, keratinocytes, released into the wound after injury

Fgf4	1450282_at	1.8	Fibroblast growth factor 4	Trophoblast proliferation and differentiation, regulation of progression through cell cycle, stem cell maintenance, embryonic limb and hindlimb morphogenesis, odontogenesis, negative regulation of apoptosis

Gata4	1441364_at	1.6	GATA binding protein 4	Embryonic development, regulation of transcription, heart development, embryonic gut morphogenesis

Gjb1	1448766_at	1.6	Gap junction membrane channel protein beta 1	Cell communication, cell-cell signaling

Kit/CD117	1452514_a_at	1.6	Kit oncogene	Germ cell development, transmembrane receptor protein tyrosine kinase signaling pathway, cell proliferation, cytokine and chemokine mediated signaling pathway, hematopoiesis, cell differentiation

Kdr	1449379_at	1.6	Kinase insert domain protein receptor	Angiogenesis, vasculogenesis, transmembrane receptor protein tyrosine kinase signaling pathway, development, cell migration, hemopoiesis, cell differentiation, cell fate commitment, endothelial cell differentiation

Nkx2-5	1449566_at	1.9	NK2 transcription factor related, locus 5	Regulation of transcription, embryonic heart tube development

Psca	1451258_at	1.5	Prostate stem cell antigen

Pou3f2	1450831_at	1.7	POU domain, class 3, transcription factor 2	Positive regulation of cell proliferation, regulation of transcription

Pou5f1/Oct-4	1417945_at	1.5	POU domain, class 5, transcription factor 1	Germ-line stem cell maintenance, expressed in mouse totipotent embryonic stem and germ cells, regulation of transcription

Sox10	1451689_a_at	2.3	SRY-box containing gene 10	Regulation of transcription, cell differentiation and maturation

Thy1/CD90	1423135_at	1.5	Thymus cell antigen 1, theta	MSC specific marker

Utf1	1416899_at	1.5	Undifferentiated embryonic cell transcription factor 1	Regulation of transcription

Col4a3bp	1420384_at	−1.6	Procollagen, type IV, alpha 3 binding protein	Goodpasture antigen binding protein

Egr2/Krox20	1427683_at	−3.9	Early growth response 2	Schwann cell differentiation, myelination, rhythmic behavior, regulates osteocalcin expression

Falz	1427310_at	−3.2	Fetal Alzheimer antigen	Negative regulation of transcription

H2-K1	1426324_at	−4.2	Histocompatibility 2, K1, K region	Immune response, antigen presentation, endogenous antigen via MHC class I

Lif	1421207_at	−8.7	Leukemia inhibitory factor (transient downregulation during cell growth)	Embryonic stem cell maintenance, immune response, tyrosine phosphorylation of Stat3 protein, muscle morphogenesis, neuron development

Lifr	1425107_a_at	−5.8	Leukemia inhibitory factor receptor	Positive regulation of cell proliferation

TNFRSF11b/OPG	1449033_aat	−34.6	Tumor necrosis factor receptor superfamily, member 11b (osteoprotegerin)	Apoptosis, signal transduction, negative regulation of osteoclastogenesis

Zfp42/Rex-1	1451244_a_at	−1.9	Zinc finger protein 42	The putative human stem cell marker, Rex-1 (Zfp42): structural classification and expression in normal human epithelial and carcinoma cell cultures

^a^Affymetrix probe ID

^b^fold change

^c^gene ontology [28]

**Table 1(b):** Genes expressed differently in PR versus P.

Gene symbol	Probe ID^a^	FC^b^	Gene Description	Biological Process^c^
Abcg2	1422906_at	−3.1	ATP-binding cassette, subfamily G, member 2	Stem cell marker, drug resistance

Cd9	1416066_at	−3.2	CD9 antigen	Stromal cell and adipose stem cell surface marker, tetraspan protein

Cd47	1419554_at	−2.4	CD47 antigen (Rh-related antigen,	Hematopoietic cells, membrane glycoprotein, the same as integrin-associated protein (IAP) and ovarian tumor marker OA3

Cd81	1416330_at	−1.6	CD 81 antigen	Cell adhesion, fertilization

Egfr	1424932_at	−1.8	Epidermal growth factor receptor	Active in early events of stem cells recruitment and differentiation

Gja7	1449094_at	−3.8	Gap junction membrane channel protein alpha 7	Cell communication, synaptic transmission, heart development, visual perception, cell development, cardiac muscle development

Il6st	1437303_at	−2.9	Interleukin 6 signal transducer	Signal transduction, positive regulation of cell proliferation, regulation of Notch signaling pathway

Lims1	1418231_at	−2.5	LIM and senescent cell antigen-like domains 1	Cell-matrix adhesion, establishment and/or maintenance of cell polarity, cell-cell adhesion, embryonic development

Cd36	1423166_at	178.8	CD36 antigen	Fatty acid transporter associated with adipogenesis

Cd200 (Ox2)	1448788_at	2.4	Cd200 antigen	Cell surface antigen of thymocytes, B cells, T cells, neurons, kidney glomeruli, tonsil follicles, the syncytiotrophoblast and endothelial cells

Cd5	1418353_at	1.6	CD5 antigen	B lymphocytes antigen

Cd63	1455777x_at	1.9	Cd63 antigen	Melanoma antigen

Vegfa	1451959_a_at	1.5	Vascular endothelial growth factor A	Regulation of progression through cell cycle, angiogenesis, development, cell proliferation, cell differentiation

Vegfb	1451803_a_at	2.6	Vascular endothelial growth factor A	Regulation of progression through cell cycle, angiogenesis, development, cell proliferation, cell differentiation

^a^Affymetrix probe ID

^b^fold change

^c^gene ontology [28]

**Table 2:** Genes regulated similarly in PR versus P and P versus V.

Gene symbol	Probe ID^a^	FC^b^		Gene description	Biological process^c^
		PR versus P	P versus V		
Akp2	1423611_at	−11.5	−2.0	Alkaline phosphatase	Marker of osteoblasts

Cd2bp2	1417224_a_at	−1.9	−1.5	CD2 antigen binding protein 2	T cell activation

Cd29 (Itgb1)	1426918_at	−2.1	−1.5	Integrin beta 1 (fibronectin receptor beta)	Regulation of progression through cell cycle, G1/S transition of mitotic cell cycle, cell adhesion, cell-matrix adhesion, integrin-mediated signaling pathway, development, positive regulation of cell proliferation, negative regulation of cell differentiation

Cd44	1423760_at	−3.9	−5.6	CD44 antigen	Cell surface glycoprotein, cell adhesion, stem cells, implicated in tumor growth and dissemination

Cd105 (Eng)	1432176_a_at	−2.3	−2.0	Endoglin	Angiogenesis, cell adhesion, heart development, regulation of transforming growth factor beta receptor signaling pathway

Cd109	1425658_at	−2.8	−5.2	CD109 antigen	Membrane glycoprotein, elevated expression in variety of cancers

H2-D1	1451934_at	−3.2	−3.0	Histocompatibility 2, D region locus 1	Immune response, detected on surface of MSC and adipocyte stem cells at low levels and reduced with passage

H2-K1	1427746_x_at	−1.6	−1.5	Histocompatibility 2, K1, K region	Immune response, antigen presentation

Mki67	1426817_at	−4.3	−5.9	Antigen identified by monoclonal antibody Ki 67	Meiosis, cell proliferation

Pcna	1417947_at	−2.4	−1.7	Proliferating cell nuclear antigen	DNA replication

S100b	1434342_at	−4.2	−2.7	S100 protein, beta polypeptide, neural	Marker of differentiated neural cells

Spred1	1460116_s_at	−1.9	−2.1	Sprouty protein with EVH-1 domain 1, related sequence	Inhibition of MAP kinases, activated in hematopoietic cells, involved in mesoderm organization, inhibit Ras pathway (G protein)

Spred2	1434403_at	−2.3	−1.7	Sprouty protein with EVH-1 domain 2, related sequence	As above

Stag1	1434189_at	−1.5	−1.7	Stromal antigen 1	Key mediator of p53-dependent apoptotic pathway, cell cycle, chromosome segregation, mitosis, and cell division

Stag2	1421849_at	−1.6	−1.6	Stromal antigen 2	As above

Cd1d1	1449130_at	4.9	5.1	CD1d1 antigen	MHC class I-like glycoprotein, development and function of natural killer T lymphocytes

Cd151	1451232_at	1.9	1.5	CD151 antigen	PPARγ positively regulates it in squamous cell carcinoma, implicated in tumor invasiveness

Fabp4	1424155_at	69.6	1.7	Fatty acid binding protein 4	Marker of differentiated adipocytes

^a^Affymetrix probe ID

^b^fold change

^c^gene ontology [28]

**Table 3:** Genes regulated differently in PR versus P and P versus V conditions.

Gene symbol	Probe ID^a^	FC^b^		Gene description	Biological process^c^
		PR versus P	P versus V		
Actc1	1415927_at	−1.5	2.0	Actin, alpha, cardiac	Cytoskeleton organization and biogenesis, muscle development, regulation of heart and muscle contraction

Actg2	1422340_a_at	−4.7	2.3	Actin, gamma 2, smooth muscle, enteric	Cytoskeleton organization and biogenesis, muscle development

Cd97	1418394_a_at	2.3	−2.1	CD97 antigen	Cell adhesion, signal transduction, G-protein coupled receptor protein signaling pathway, neuropeptide signaling pathway

Cd166 (ALCAM)	1437466_at	2.1	−1.5	Activated leukocyte cell adhesion molecule	Cell adhesion, axon guidance, motor axon guidance

Cxcl1	1419209_at	−2.7	1.8	Chemokine (C-X-C motif) ligand 1	Regulation of progression through cell cycle, inflammatory response, immune response

Cxcl4	1448995_at	−2.1	2.8	Chemokine (C-X-C motif) ligand 4	Chemotaxis, immune response, negative regulation of angiogenesis, cytokine, and chemokine mediated signaling pathway, platelet activation, negative regulation of megakaryocyte differentiation

Cxcl12 (SDF-1)	1417574_at	−2.4	7.5	Chemokine (C-X-C motif) ligand 12 (stem cell differentiation factor)	Patterning of blood vessels, ameboidal cell migration, chemotaxis, immune response, germ cell development and migration, brain development, motor axon guidance, T cell proliferation, induction of positive chemotaxis

Cxcl16	1456428_at	−1.7	1.7	Chemokine (C-X-C motif) ligand 15	Chemotaxis, inflammatory response, immune response, signal transduction, hematopoiesis, neutrophil chemotaxis

Foxa1	1418496_at	−1.5	1.9	Forkhead box A1	Regulation of transcription, lung development, epithelial cell differentiation, branching morphogenesis of a tube

Kitl	1415854_at	−4.1	5.2	Kit ligand	Cell adhesion, germ cell development, positive regulation of peptidyl-tyrosine phosphorylation, cytokine product associated with MSC/stromal cells, stem cell factor

Ntf3	1450803_at	−1.5	1.9	Neurotrophin 3	Neuromuscular synaptic transmission, glial cell fate determination, axon guidance, brain and peripheral nervous system development, epidermis development, mechanoreceptor differentiation, regulation of neuron apoptosis

Pdgfα	1421916_at	−2.1	1.6	Platelet derived growth factor receptor, alpha polypeptide	Protein amino acid phosphorylation, transmembrane receptor protein tyrosine kinase signaling pathway, morphogenesis, organ morphogenesis, extracellular matrix organization and biogenesis, male genitalia development, odontogenesis

Tnfsf11 (RANKL)	1419083_at	−1.6	9.2	Tumor necrosis factor (ligand) superfamily, member 11	Positive regulation of osteoclast differentiation and bone resorption, immune response, lymph node development

Snai2	1418673_at	−6.4	1.9	Snail homolog 2 (Drosophila)	Development of human melanocytes, regulation of transcription, DNA dependent, development, response to radiation, regulation of survival gene product activity

Vegfc	1419417_at	−5.6	11.5	Vascular endothelial growth factor C	Regulation of progression through cell cycle, angiogenesis, positive regulation of neuroblast proliferation, development, positive regulation of cell proliferation, organ morphogenesis

^a^Affymetrix probe ID

^b^fold change

^c^gene ontology [28]

**Table 4:** Genes whose expression was not affected in P versus V and PR versus P conditions.

Gene symbol	Probe ID^a^	Gene description
Afp	1416645_a_at	Alpha fetoprotein
Cd34	1416072_at	CD34 antigen
Cd3z	1438392_at	CD3 antigen, zeta polypeptide
Cd5l	1449193_at	CD5 antigen like
Cd6	1451910_a_at	CD6 antigen
Cd8b1	1448569_at	CD8 antigen, beta chain 1
Cd22	1419769_at	CD22 antigen
Cd53	1439589_at	CD53 antigen
Cd86	1420404_at	CD86 antigen
Cd164	1431527_at	CD164 antigen
Cd209e	1420582_at	Cd209e antigen
Cdh15	1418602_at	Protocadherin 15
Cer1	1450257_at	Cerberus 1 homolog
Col6a2	1452250_a_at	Procollagen, type VI, alpha 2
Erbb2ip	1439080_at	Erbb2 interacting protein
Erbb3	1452482_at	V-erb-b2 erythroblastic leukemia viral oncogene homolog 3 (avian)
Fabp7	1450779_at	Fatty acid binding protein 7, brain
Fzd9	1427529_at	Frizzled homolog 9
Gata2	1450333_a_at	GATA binding protein 2
Gcg	1425952_a_at	Glucagon
Gcm2	1420455_at	Glial cells missing homolog 2
Gfap	1440142_s_at	Glial fibrillary acidic protein
Gjb3	1416715_at	Gap junction membrane channel protein beta 3
Gjb4	1422179_at	Gap junction membrane channel protein beta 4
Ina	1418178_at	Internexin neuronal intermediate filament protein, alpha
Ins1	1422447_at	Insulin I
Isl1	1444129_at	ISL1 transcription factor, LIM/homeodomain (islet 1)
Krt1-14	1460347_at	Keratin complex 1, acidic, gene 14
Krt1-17	1423227_at	Keratin complex 1, acidic, gene 17
Krt2-8	1435989_x_at	Keratin complex 2, basic, gene 8
Mbp	1454651_x_at	Myelin basic protein
Mtap1b	1450397_at	Microtubule-associated protein 1 B
Myh11	1448962_at	Myosin, heavy polypeptide 11, smooth muscle
Ncam1	1439556_at	Neural cell adhesion molecule 1
Ncam2	1425301_at	Neural cell adhesion molecule 2
Nes	1453997_a_at	Nestin
Ngfr	1421241_at	Nerve growth factor receptor (TNFR superfamily, member 16)
Nkx2-2	1421112_at	NK2 transcription factor related, locus 2 (Drosophila)
Numb	1425368_a_at	Numb gene homolog (Drosophila)
Olig1	1416149_at	Oligodendrocyte transcription factor 1
Pax6	1456342_at	Paired box gene 6
Pou3f3	1422331_at	POU domain, class 3, transcription factor 3
Pou6f1	1420749_a_at	POU domain, class 6, transcription factor 1
Prox1	1457432_at	Prospero-related homeobox 1
Ptprc	1440165_at	Protein tyrosine phosphatase, receptor type, C
Slc1a2	1451627_a_at	Solute carrier family 1 (glial high affinity glutamate transporter), member 2
Slc1a6	1418933_at	Solute carrier family 1 (high affinity aspartate/glutamate transporter), member 6
Sox1	1422205_at	SRY-box containing gene 1
Sox2	1416967_at	SRY-box containing gene 2
Syn1	1453467_s_at	Synapsin I
Tubb3	1415978_at	Tubulin, beta 3
Zfp110	1450998_at	Zinc finger protein 110

^a^Affymetrix probe ID
